# Which prognostic factors for recurrence after transanal endoscopic microsurgery for early rectal cancer?

**DOI:** 10.1007/s00464-026-12632-9

**Published:** 2026-02-18

**Authors:** Alberto Arezzo, Carlo Alberto Ammirati, Giovanni Distefano, Michele Barbiero, Francesca Sbuelz, Roberto Passera, Mario Morino

**Affiliations:** 1 Department of Surgical Sciences, University of Turin, Corso Dogliotti 14, 10126 Turin, Italy; 2 Biostatistics Unit, Department of Medical Sciences, University of Turin, Turin, Italy

**Keywords:** Transanal endoscopic microsurgery, TEM, Hermanek criteria, Rectal neoplasms, Local excision, Prognostic factors, Recurrence

## Abstract

**Background:**

Transanal endoscopic microsurgery (TEM) enables the local excision of rectal neoplasms with minimal invasiveness; however, the long-term oncologic safety for pTis and pT1 lesions, as well as the significance of traditional histopathological risk factors, remains controversial.

**Methods:**

A retrospective cohort of 170 consecutive patients undergoing en bloc, R0 full-thickness TEM for rectal pTis or pT1 adenocarcinoma between 1994 and 2025 at a single tertiary centre was analysed. Pathological review included Vienna classification, submucosal invasion (sm1 vs sm2–3), tumour grade (G1–2 vs G3), lymphovascular invasion (LVI), mucinous histotype, tumour budding, perineural invasion (PNI), and tumour diameter (> 3 cm). Patients with adverse features were recommended salvage radical surgery or adjuvant radiotherapy. Disease-free (DFS) and overall survival (OS) were estimated using Kaplan–Meier analysis and log-binomial regression.

**Results:**

The median age was 71 years (IQR, 62–77), and the median follow-up was 24 months (IQR, 24–53). Lesion distribution was Vienna 4.2 (*n* = 21), Vienna 4.4 (*n* = 21), pT1sm1 (*n* = 48), pT1sm2 (*n* = 40), and pT1sm3 (*n* = 40). All patients achieved R0 excision. No recurrences were observed in the Vienna 4.2/4.4 groups. For pT1 tumours, recurrence rates were 4.2% (sm1), 37.5% (sm2), and 20.0% (sm3). On multivariate analysis, submucosal invasion (sm2–3; HR 5.67, p = 0.021), high grade (G3; HR 3.58, *p* = 0.004), and LVI (HR 3.58, *p* = 0.003) were independent predictors of DFS. Other factors were not significant. In OS analysis, older age (> = 71 years) and tumour diameter > 3 cm were associated with poorer survival, whereas classical high-risk pathological factors (sm2–3, G3, LVI) were not.

**Conclusion:**

TEM affords excellent long-term disease control for Vienna 4.2/4.4 lesions. In pT1 adenocarcinoma, only submucosal invasion beyond sm1, high grade, and LVI independently predict recurrence, underscoring the need for risk-adapted, multidisciplinary management.

**Supplementary Information:**

The online version contains supplementary material available at 10.1007/s00464-026-12632-9.

Transanal endoscopic microsurgery (TEM) was developed between 1980 and 1983 by Gerhard Buess with the primary goal of avoiding radical surgery in patients affected by large laterally spreading adenomas [1]. This novel endoscopic technique, which provided a stable pneumorectum and high-definition, magnified visualisation, allowed for the precise full-thickness excision of rectal lesions greater than 2 cm in diameter with clear margins.

During the initial clinical experience, Buess observed that approximately 10% of these large adenomas were found to be malignant tumours limited to the submucosal layer—i.e. pathological T1 adenocarcinomas [2]. At that time, the prevailing oncological policy mandated a second-stage radical surgery with total mesorectal excision and regional lymphadenectomy in all cases with unexpected carcinoma. However, not all patients accepted such completion surgery. In a subsequent retrospective analysis published in 1992, Buess reported that the risk of disease recurrence in selected patients treated with local excision alone was surprisingly low [3].

This clinical insight was aligned with the histopathological criteria, which identified three key risk factors for lymph node metastasis in T1 rectal cancers: (1) depth of submucosal invasion over sm1, (2) poor tumour differentiation (G3), and (3) presence of lymph vascular invasion (LVI) [4]. Over the years, these criteria have been re-evaluated and challenged. The so-called Paris classification, although based on limited data, redefined sm1 invasion as a tumour extension of ≤ 1 mm beyond the muscularis mucosae, rather than the upper third of the submucosa [5]. This definition aimed to identify cases with a lymph node metastasis risk < 1% and to allow proper staging of specimens resected within the submucosal plane, where the full thickness of the submucosa cannot be reliably assessed. More recently, additional histological markers have emerged, such as tumour budding, while the independent prognostic value of submucosal depth alone has been questioned. A large pooled meta-analysis published last year challenged the notion that invasion depth is a dominant risk factor in the absence of other adverse features [6].

In this context, we sought to reevaluate our institutional series of pTis and pT1 rectal adenocarcinomas treated with TEM, reassessing the prognostic relevance of classical histopathological risk factors in predicting overall survival (OS) and disease-free survival (DFS).

## Materials and methods

### Study design and patient selection

This retrospective cohort study was conducted at the General Surgery Unit I, University of Turin, Città della Salute e della Scienza, Italy. All consecutive patients undergoing full-thickness local excision of rectal neoplasms via transanal endoscopic microsurgery (TEM) between 15 November 1994 and 1 April 2025 were considered for inclusion. The study cohort was drawn from a prospectively maintained institutional database that continuously recorded surgical, oncological, and pathological parameters for all patients treated with TEM over 30 years.

Eligible patients included those with a final pathological diagnosis of either carcinoma in situ (Tis) or early invasive adenocarcinoma (pT1) according to AJCC/UICC TNM 8th edition. The indication for local excision was either preoperative suspicion of a benign lesion or presumed early rectal cancer assessed as low risk. Preoperative assessment was coordinated by colorectal surgeons in collaboration with dedicated endoscopists and radiologists and included high-resolution endoscopy, digital rectal examination, and routine endorectal ultrasound (transanal ultrasound) for local T staging. Pelvic MRI was used for the assessment of mesorectal involvement and nodal status when available; as imaging practice evolved over the 30-year period, MRI progressively became the standard cross-sectional modality in the later years.

Exclusion criteria were as follows:

1. Non-adenocarcinoma histology (e.g. neuroendocrine tumours, squamous cell carcinoma, GIST).
2. Preoperative (neo)adjuvant therapy such as (chemo)radiotherapy.
3. Synchronous advanced colorectal cancer.
4. Incomplete or missing pathological records precluding accurate classification or staging.

All patients provided written informed consent for both the procedure and data collection. The study was conducted in accordance with institutional regulations and the Declaration of Helsinki. Local ethics committee approval was waived, given the retrospective and anonymised nature of the analysis.

### Data collection and histopathologic classification

Clinical data, including demographics, comorbidities, preoperative workup, surgical details, histopathology, and follow-up, were extracted from the prospectively maintained institutional TEM database.

Histologic staging adhered to the 8th edition of the AJCC/UICC TNM classification. For Tis lesions, classification was refined using the Vienna Classification of Gastrointestinal Epithelial Neoplasia [7], which defines:

- Category 4.2: Non-invasive high-grade neoplasia (carcinoma in situ),
- Category 4.4: Intramucosal adenocarcinoma.

For pT1 lesions, the following factors were extracted from the pathology reports:

- 1. Depth of submucosal invasion, initially reported in thirds (sm1/sm2/sm3), and reclassified according to the Paris classification [5] as:
  - sm1: invasion ≤ 1 mm from the muscularis mucosae (upper third),
  - sm2–3: invasion > 1 mm (middle to deep thirds),

2. Tumour grade, with G3 (poorly differentiated) considered high-risk,
3. Lymphovascular invasion (LVI),
4. Mucinous histotype, defined as tumours with > 50% mucin content,
5. Tumour budding, recorded when small clusters (< 5 cells) were seen at the invasive front,
6. Perineural invasion (PNI),
7. Maximum tumour diameter, recorded in centimetres.

Experienced gastrointestinal pathologists reviewed all pathology specimens, and data were centrally curated to ensure consistency.

Patients whose pathological reports identified one or more high-risk features (deep submucosal invasion sm2-3, G3, LVI +) were offered radical oncologic surgery (e.g. low anterior resection or abdominoperineal resection with total mesorectal excision). For patients refusing further surgery or deemed unfit for radical intervention, adjuvant radiotherapy was offered when appropriate.

Chemoradiotherapy or systemic therapy was reserved for recurrence, per national and international guidelines (AIOM, ESMO). A multidisciplinary board made all therapeutic decisions.

### Follow-up protocol and oncologic management

Follow-up was conducted according to institutional guidelines, which included:

- physical examinations every 3 months for the first 3 years, then every 6 months, including digital exploration.
- tumour markers (CEA) titers every 3 months for the first 3 years, then every 6 months.
- flexible endoscopic surveillance by rectosigmoidoscopy every 6 months for the first 3 years.
- CT scan of chest and abdomen annually for 5 years, or as clinically indicated.

### Outcomes and statistical analysis

The primary outcomes were as follows:

- Disease-free survival (DFS), defined as the time from TEM to the first documented recurrence (local or distant).

- Overall survival (OS), defined as the time from TEM to death from any cause.

Survival curves were estimated by the Kaplan–Meier method and compared across groups by the log-rank test. For pT1 cancer patients, the effect on OS and DFS of the same set of risk factors has been estimated using uni- and multivariate Cox proportional hazards regression models, comparing the two arms with the Wald test and calculating 95% confidence intervals (CI). Patient characteristics were analysed using Fisher’s exact test for categorical variables and the Mann–Whitney and Kruskal–Wallis tests for continuous variables; continuous covariates were described as median/inter quartile range (IQR). All reported p-values were obtained by the two-sided exact method at the conventional 5% significance level. Data were analysed as of August 2025 by R 4.5.1 (R Foundation for Statistical Computing, Vienna-A, http://www.R-project.org). A p-value < 0.05 was considered statistically significant. To address potential temporal and therapeutic heterogeneity, we performed sensitivity analyses in pT1 patients by (i) adding surgery period (< 2010 vs ≥ 2010) as a covariate in the multivariable Cox model, and (ii) including post-TEM management (completion radical surgery and/or adjuvant radiotherapy) as additional covariates.

## Results

### Patient population and histopathologic characteristics

Between 15 November 1994 and 1 April 2025, a total of 170 patients underwent full-thickness transanal local excision using transanal endoscopic microsurgery (TEM) for either carcinoma in situ (pTis, 42 patients) or early invasive rectal adenocarcinoma (pT1, 128 patients) at the Department of Surgery, General Surgery Unit I, University of Turin, Italy. All procedures were performed with curative intent. Until 2014, surgery was routinely performed under general anaesthesia. From 2015 onward, spinal anaesthesia became the preferred approach, consistent with evidence supporting its safety and feasibility [8].

Two experienced colorectal surgeons performed all procedures using a standardised TEM platform (Richard Wolf, Knittingen, Germany) until 2008, followed by the Transanal Endoscopic Operation (TEO) platform (Karl Storz, Tuttlingen, Germany).

Table 1 shows the characteristics of the study cohort. The final histopathologic assessment revealed that 21 patients (12.3%) had Vienna 4.2 lesions, 21 patients (12.3%) had Vienna 4.4 lesions, 48 patients (28.2%) had pT1sm1 tumours, 40 patients (23.5%) had pT1sm2 tumours, and 40 patients (23.5%) had pT1sm3 tumours. The median age was 71 years (IQR 62–77). All patients underwent R0 full-thickness excision. The median follow-up for the overall cohort was 24 months (IQR 24–53), reflecting the predominance of cases treated in the last decade. Tumour grade and other invasive cancer risk features (e.g. LVI) are reported for pT1 tumours only (*n* = 128), as they are not applicable to non-invasive Vienna 4.2/4.4 lesions.

**Table 1:** Baseline characteristics and pathology (overall cohort; pT1-only variables as specified)

Variable	Category	Value
Age (years)	Median (IQR)	71 (62–77)
Gender	Male / Female	112 / 58
Tumour diameter (cm)	Median (IQR)	3 (2–4)
Distal tumour margin from anal verge (cm)	Median (IQR)	6 (4–8)
Follow-up (months)	Median (IQR)	24 (24–53)
Histologic group		
	Vienna 4.2	21 (12.4%)
	Vienna 4.4	21 (12.4%)
	pT1sm1	48 (28.2%)
	pT1sm2	40 (23.5%)
	pT1sm3	40 (23.5%)
Tumour grade	pT1 only (*n* = 128)	
	G1	25 (19.5%)
	G2	92 (71.9%)
	G3	11 (8.6%)
Lymphovascular invasion (LVI)	pT1 only (*n* = 128)	
	No	114 (89.1%)
	Yes	14 (10.9%)
Perineural invasion (PNI)	pT1 only (*n* = 128)	
	No	124 (96.9%)
	Yes	4 (3.1%)
Mucinous histotype	pT1 only (*n* = 128)	
	No	120 (93.8%)
	Yes	8 (6.2%)
High-grade tumour budding	pT1 only (*n* = 128)	
	No	112 (87.5%)
	Yes	16 (12.5%)
Positive resection margin	pT1 only (*n* = 128)	
	No	114 (89.1%)
	Yes	14 (10.9%)

Grading, LVI, PNI, mucinous histotype, tumour budding, and margin status were evaluated for pT1 cancers; they are not applicable to Vienna 4.2/4.4 lesions and therefore are presented for pT1 patients only

To address potential temporal heterogeneity over the 30-year period, we performed a sensitivity analysis in pT1 patients comparing cases treated before 2010 versus 2010 onwards (Supplementary Table S4). Recurrence rates were 14.3% (7/49) in the pre-2010 cohort and 22.8% (18/79) in the 2010 + cohort, and the direction and magnitude of the associations for sm2-3, G3, and LVI were consistent. When period (2010 + vs pre-2010) was added to the multivariable Cox model for DFS (adjusted for sm2-3, G3, LVI, completion surgery and adjuvant radiotherapy), period was not independently associated with recurrence (HR 1.79, 95% CI 0.67–4.77; *p* = 0.24).

Among pT1 patients, 85 (66.4%) had at least one classic high-risk feature (submucosal invasion beyond sm1, G3 differentiation and/or LVI) and were recommended completion radical surgery with total mesorectal excision. Completion surgery was undertaken in 17 patients (13.3% of pT1); in three cases, residual tumour and/or nodal metastases were identified. Sixty-eight patients declined further surgery or were deemed unfit; of these, 9 received adjuvant short-course radiotherapy (5 × 5 Gy).

### Oncologic outcomes and follow-up

During follow-up, no recurrences were observed in patients with Vienna 4.2 or Vienna 4.4 lesions (0 of 21 in each group). Among patients with pT1sm1 tumours, 2 of 48 (4.2%) experienced recurrence, both local, and none developed distant metastases. In the pT1sm2 group, 15 out of 40 patients (37.5%) experienced recurrence, with 13 (32.5%) presenting with local recurrence and 5 (12.5%) with distant metastases. For pT1sm3 tumours, 8 of 40 patients (20.0%) developed recurrence, all of which were local except for one patient (2.5%) who had distant metastases. The lung was the most frequent site of distant recurrence, followed by the liver.

Overall, among pT1 tumours, 25 recurrences occurred: 19 were local-only, 2 distant-only, and 4 combined local and distant. The median time to recurrence was 15 months (IQR 9–22).

### Survival analysis by risk features

**Fig. 1:**
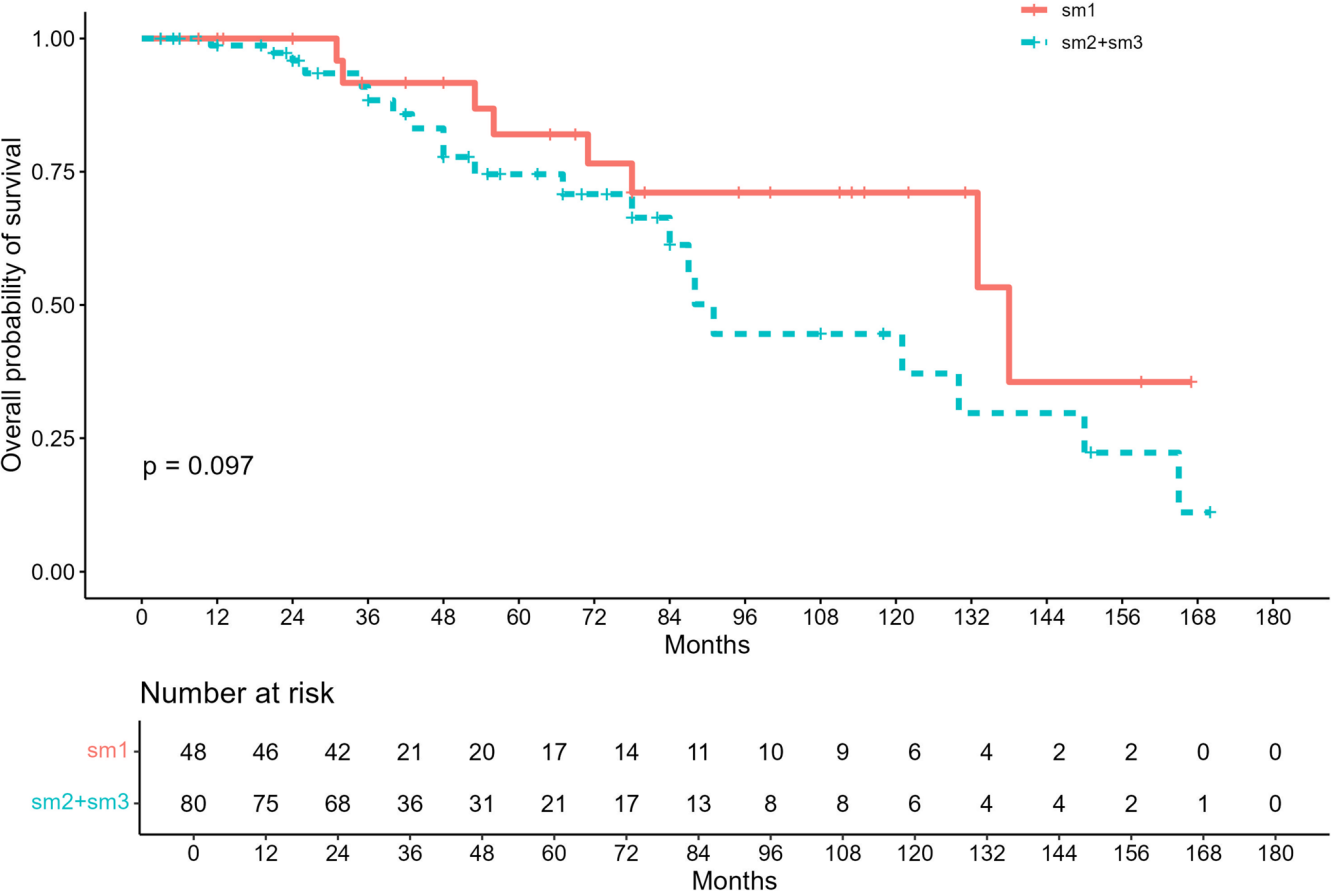
Kaplan–Meier overall survival (OS) for pT1 rectal adenocarcinoma stratified by depth of submucosal invasion (sm1 vs sm2–3). Numbers at risk shown

**Fig. 2:**
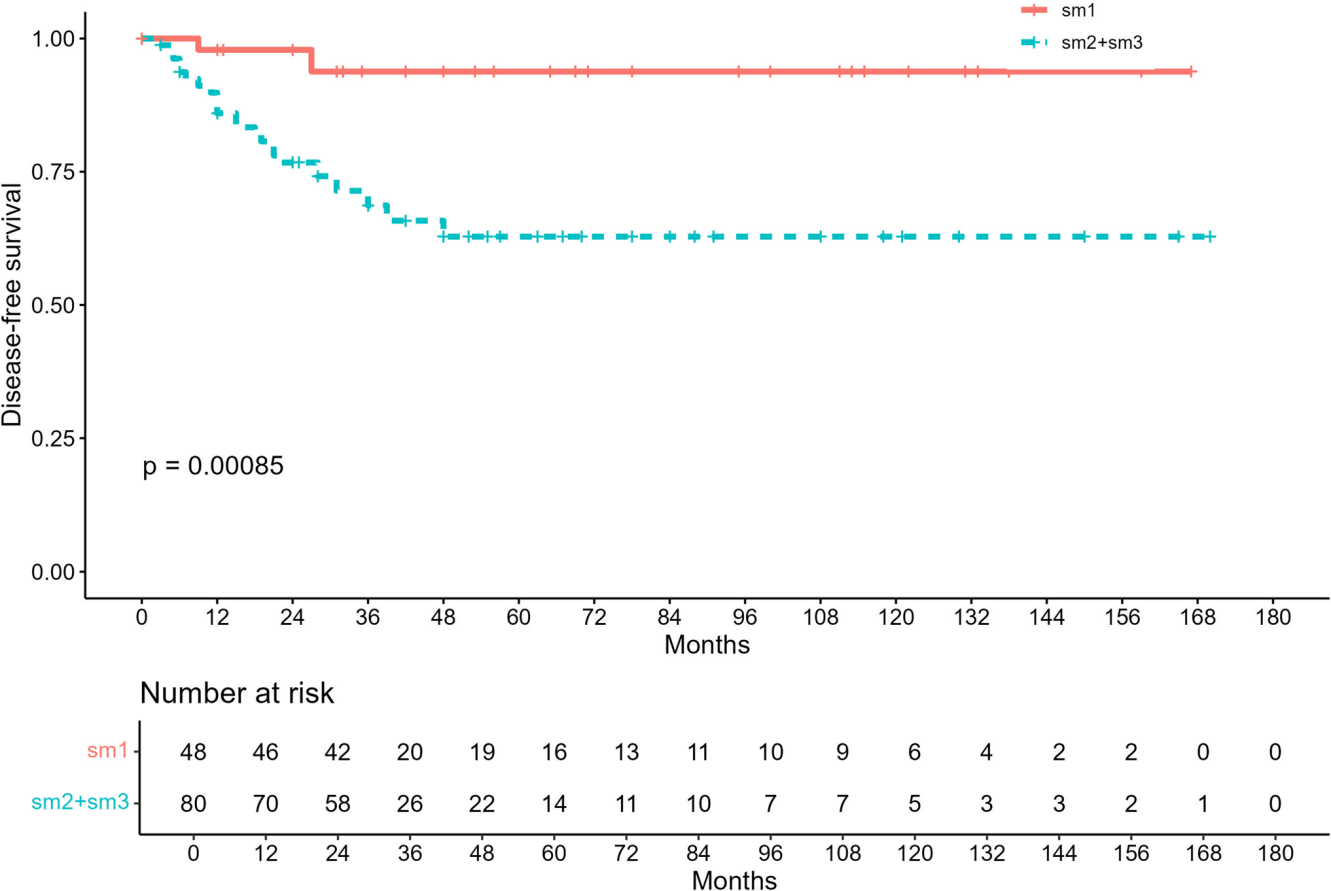
Kaplan–Meier disease-free survival (DFS) for pT1 rectal adenocarcinoma stratified by depth of submucosal invasion (sm1 vs sm2-3). Numbers at risk shown

Kaplan–Meier curves for OS by depth of submucosal invasion (sm1 vs sm2–3) did not demonstrate significantly worse survival in patients with sm2–3 lesions (Fig. 1), while a statistical difference emerged for DFS at K–M curves (Fig. 2).

For pT1 cancers only, univariate and multivariate analyses were performed using uni- and multivariate Cox proportional hazards regression (HR) models with 95% confidence intervals.

Table 2 shows the univariate analyses for OS. None of the investigated pathological variables demonstrated a statistically significant association with patient outcomes, except the age of the patient at index surgery. Specifically, submucosal invasion beyond sm1 (sm2–3) was not significantly related to overall survival (OS) (HR 1.97, 95% CI 0.87–4.48; p = 0.104). Similarly, high tumour grade (G3), lymphovascular invasion, perineural invasion, mucinous histotype, high tumour budding, and tumour diameter greater than 3 cm all failed to reach statistical significance (all p > 0.2). These findings indicate that, within this cohort, no single pathological factor independently predicted overall survival (OS) following local excision of pT1 rectal cancer. A multivariate analysis was therefore not performed.

**Table 2:** Overall survival (OS)–univariate Cox regression in pT1 cancers (*n* = 128)

Covariate	HR (95% CI)	p
Age (≥ 71 vs ≤ 70 years)	2.67 (1.23–5.77)	0.013
Gender (F vs M)	0.82 (0.37–1.81)	0.628
Submucosal invasion (sm2–3 vs sm1)	1.97 (0.87–4.47)	0.104
Tumour budding (high vs low grade)	1.18 (0.44–3.15)	0.741
Lymphovascular invasion (yes vs no)	1.04 (0.36–3.05)	0.936
Perineural invasion (yes vs no)	0.44 (0.06–3.36)	0.431
Mucinous histotype (yes vs no)	0.89 (0.21–3.78)	0.875
Tumour grade (G3 vs G1–2)	1.44 (0.50–4.17)	0.499
Tumour diameter (> 3 cm vs ≤ 3 cm)	2.51 (1.19–5.33)	0.016
Completion (salvage) radical surgery (yes vs no)	0.81 (0.19–3.48)	0.780
Adjuvant short-course radiotherapy (yes vs no)	0.60 (0.18–2.00)	0.401

In exploratory models accounting for post-TEM management, neither completion surgery nor adjuvant radiotherapy showed a statistically significant association with DFS, and the hazard ratios for sm2–3, G3, and LVI remained materially unchanged.

Table 3 shows the univariate and multivariate analyses for DFS. Through multivariate analysis, several pathological features were identified as independently associated with disease-free survival. Specifically, submucosal invasion beyond sm1 (sm2–3) was a strong predictor of recurrence (HR 5.67, 95% CI 1.30–24.81; *p* = 0.021). High tumour grade (G3) was also associated with a significantly increased risk of recurrence compared to low-to-intermediate grade tumours (HR 3.58, 95% CI 1.51–8.49; *p* = 0.004). Furthermore, the presence of lymphovascular invasion was independently associated with a higher risk of recurrence (HR 3.58, 95% CI 1.54–8.32; *p* = 0.003).

**Table 3:** Disease-free survival (DFS)–univariate and multivariate Cox regression in pT1 cancers (*n* = 128)

Covariate	Univariate HR (95% CI)	Univariate p	Multivariate HR (95% CI)	Multivariate p
Age (≥ 71 vs ≤ 70 years)	0.65 (0.29–1.46)	0.294	–	–
Gender (F vs M)	0.77 (0.32–1.84)	0.555	–	–
Submucosal invasion (sm2–3 vs sm1)	7.91 (1.86–33.56)	0.005	5.51 (1.24–24.54)	0.025
Tumour budding (high vs low grade)	1.17 (0.40–3.42)	0.771	–	–
Lymphovascular invasion (yes vs no)	4.02 (1.73–9.34)	0.001	3.48 (1.48–8.18)	0.004
Perineural invasion (yes vs no)	1.25 (0.17–9.29)	0.825	–	–
Mucinous histotype (yes vs no)	5.24 (1.96–14.03)	< 0.001	–	–
Tumour grade (G3 vs G1–2)	5.84 (2.52–13.57)	< 0.001	3.59 (1.50–8.61)	0.004
Tumour diameter (> 3 cm vs ≤ 3 cm)	1.20 (0.53–2.72)	0.668	–	–
Completion (salvage) radical surgery (yes vs no)	1.73 (0.65–4.62)	0.273	1.23 (0.45–3.40)	0.684
Adjuvant short-course radiotherapy (yes vs no)	1.67 (0.50–5.58)	0.405	1.40 (0.40–4.89)	0.602

The multivariate model includes the classic pathological risk factors (sm2–3, G3, LVI) and post-TEM management (completion surgery and adjuvant radiotherapy) to address potential therapeutic confounding; treatment covariates were not statistically significant and did not materially change the pathological hazard ratios

Separate analyses were performed for the subgroup of Vienna 4.2 vs. 4.4 lesions, evaluating their impact on DFS and OS using RR models and survival curves. When comparing patients with Vienna 4.2 and Vienna 4.4 lesions, no disease recurrences were observed in either group during follow-up, resulting in a disease-free survival (DFS) rate of 100% for both cohorts. Similarly, overall survival (OS) did not differ significantly between the two groups, with no disease-related deaths reported.

## Discussion

This retrospective analysis provides robust evidence regarding the long-term oncologic safety and risk stratification of transanal endoscopic microsurgery (TEM) for the treatment of early rectal neoplasia, including both carcinoma in situ and intramucosal carcinoma (Vienna 4.2 and 4.4) and pT1 invasive adenocarcinoma. Our findings offer new insights into patient selection, the prognostic value of histopathologic risk factors, and the clinical outcomes associated with organ-preserving local excision in this context. When treating even superficial rectal neoplasms, it is imperative to achieve en bloc excision with histologically negative margins in 100% of cases. This approach not only guarantees oncologic safety but also enables a reliable pathological assessment of risk factors without confounding variables related to suboptimal resection technique, as may occur with endoscopic resections such as endoscopic mucosal resection (EMR) or endoscopic submucosal dissection (ESD).

One of the most salient findings of this study is the remarkable oncologic safety of TEM in the management of Vienna 4.2 and 4.4 lesions. In our series, neither group experienced any local or distant recurrence throughout long-term follow-up, and both achieved a disease-free survival (DFS) rate of 100%. These results strongly support the low-risk nature of these lesions, suggesting that, in the absence of invasive features or adverse histology, up to intramucosal carcinoma may be safely managed with organ-sparing approaches [7]. The equivalence in outcome between Vienna 4.2 (carcinoma in situ) and Vienna 4.4 (intramucosal carcinoma with glandular crowding) further supports the rationale for grouping these lesions in risk-adapted management protocols [9, 10].

In contrast, the analysis of pT1 tumours reveals a distinct subset of patients at significantly higher risk for recurrence, depending on pathological features. Our multivariate analysis identified three independent predictors of disease recurrence after local excision of pT1 rectal adenocarcinoma: depth of submucosal invasion (sm2–3), high histologic grade (G3), and the presence of lymphovascular invasion (LVI). These findings align with the classic risk stratification first described many years ago and supported by subsequent large series [9, 10]. The clinical significance of deep submucosal invasion as a predictor of lymph node metastasis has recently been questioned by Zwager et al. In a comprehensive meta-analysis including 67 studies and over 21,000 patients, deep submucosal invasion was associated with a higher risk of lymph node metastasis in univariate analysis (OR 2.58; 95% CI, 2.10–3.18). However, when analysed in a multivariable context (8 studies, 3621 patients), deep submucosal invasion did not remain a statistically significant independent predictor for nodal involvement (OR 1.73; 95% CI, 0.96–3.12) [6], while poor differentiation (OR 2.14), high-grade tumour budding (OR 2.83), and lymphovascular invasion (OR 3.16) were all confirmed. Importantly, the median follow-up was 24 months, largely because 55% of patients were treated since 2015 and 36% since 2020; therefore, late events may be under-represented in a proportion of cases and OS comparisons should be interpreted cautiously.

On the contrary, our data reinforce the principle that not all pT1 lesions are equal in terms of recurrence risk following local excision. The strong association between deep submucosal invasion, the presence of LVI, and high tumour grading with disease recurrence in our series is well-established adverse features that portend a worse prognosis [11, 12]. Their inclusion in modern guidelines as indications for completion radical surgery is well justified by our data, which demonstrate their independent prognostic impact in a contemporary, prospectively maintained cohort. Conversely, other factors—such as perineural invasion, mucinous histotype, tumour budding, and tumour size greater than 3 cm—were not independently associated with DFS in our multivariate analysis. While some reports have suggested a potential role for these features in risk stratification [13–15], our findings suggest their prognostic utility is limited in the context of pT1 rectal cancer managed by local excision.

Notably, none of the investigated pathological factors showed a statistically significant association with overall survival (OS) in this cohort. While adverse features such as deep submucosal invasion, high grade, or LVI increased the risk of disease recurrence, this did not translate into a detectable difference in OS. There are several plausible explanations for this finding. First, the overall number of cancer-related deaths in early-stage rectal cancer after local excision is low. Second, the relatively short follow-up in some patients and the competing risk of death from unrelated causes may limit the statistical power to detect survival differences. Ultimately, treatment pathways following local excision were highly heterogeneous, reflecting also patient preferences. Many patients declined radical completion surgery despite its recommendation, mainly due to concerns about functional morbidity and impaired quality of life following radical rectal resection.

Another key confounder is the use of radiotherapy as adjuvant treatment in patients undergoing local excision. It is now well established, based on the results of large, randomised trials such as STAR-TREC and GRECCAR 2, that neoadjuvant radiotherapy can enable organ-preserving, transanal local excision for a substantial proportion of patients with early rectal cancer, offering comparable oncologic safety to radical surgery [13–15]. More recently, interim results from the TESAR trial have confirmed a possible significant role for adjuvant radiotherapy after local excision, demonstrating a reduction in local recurrence for high-risk early rectal cancers [16]. These developments highlight the rapidly evolving landscape of multidisciplinary management and the critical need for individualised, patient-centred treatment planning after local excision.

The identification of sm2–3 invasion, G3, and LVI as independent risk factors for DFS mirrors the recommendations of leading guidelines, including the American Society of Colon and Rectal Surgeons (ASCRS) and the European Society for Medical Oncology (ESMO), which endorse completion radical surgery for pT1 lesions with adverse features [17, 18]. Notably, our results do not support the use of perineural invasion, mucinous histotype, tumour budding, or tumour size greater than 3 cm as sole indications for radicalisation, in agreement with several recent pooled analyses [19, 20]. Only relevant improvements in intraoperative staging of rectal cancer at the time of local excision, such as the use of fluorescence to better characterise tumour histology and invasiveness [21], or sampling of potential sentinel lymph nodes in the mesorectum [22], could significantly alter the perspective on organ-sparing techniques.

The strengths of this study include the large, single-institution cohort with prospectively collected data, uniform surgical technique by experienced colorectal surgeons, and long-term follow-up. The use of standardised pathologic criteria and multivariate modelling enhances the reliability and applicability of the findings. Limitations include the retrospective design and the inherent potential for selection bias, especially in decisions regarding salvage surgery or adjuvant treatment. The modest sample size, particularly for subgroups with high-risk features, may limit statistical power for rare outcomes such as disease-specific mortality. Finally, as with all observational studies, unmeasured confounding cannot be excluded.

In conclusion, this study demonstrates the excellent long-term oncologic outcomes of TEM for Vienna 4.2 and 4.4 lesions, as well as the critical importance of established pathologic risk factors—submucosal invasion beyond sm1, high tumour grading, and LVI—for recurrence in pT1 rectal adenocarcinoma. No pathological variable independently predicted overall survival (OS), reinforcing the effectiveness of surveillance and salvage in contemporary practice. These results strongly support a risk-adapted, organ-preserving approach for early rectal cancer and highlight the necessity for careful multidisciplinary management [23]. Future studies, ideally with larger multi-institutional cohorts and detailed quality-of-life assessments, are warranted to refine indications further and optimise patient selection for local excision.

## Supplementary Information

Below is the link to the electronic supplementary material.

Supplementary file1 (DOCX 29 KB)
