# Supplementary material for: Which prognostic factors for recurrence after transanal endoscopic microsurgery for early rectal cancer?
Source: Surg Endosc. 2026 Feb 18;40(5):3729–37. doi: 10.1007/s00464-026-12632-9 (PMC13161008; doi:10.1007/s00464-026-12632-9)
Supplement: Supplementary file 1 — Supplementary file1 (DOCX 29 KB) [file 464_2026_12632_MOESM1_ESM.docx]

**Supplementary Material**

**Supplementary Table S1. Pathological features, management, and recurrence by submucosal invasion stage (pT1 only).**

| Characteristic | sm1 (n=48) | sm2 (n=40) | sm3 (n=40) |
| --- | --- | --- | --- |
| G3 (poor differentiation) | 0 (0.0%) | 7 (17.5%) | 4 (10.0%) |
| Lymphovascular invasion (LVI+) | 5 (10.4%) | 6 (15.0%) | 3 (7.5%) |
| Mucinous histotype | 0 (0.0%) | 5 (12.5%) | 3 (7.5%) |
| Perineural invasion (PNI+) | 1 (2.1%) | 2 (5.0%) | 1 (2.5%) |
| High-grade tumour budding | 1 (2.1%) | 6 (15.0%) | 9 (22.5%) |
| Diameter >3 cm | 16 (33.3%) | 17 (42.5%) | 13 (32.5%) |
| Positive margin | 2 (4.2%) | 4 (10.0%) | 8 (20.0%) |
| Completion surgery | 2 (4.2%) | 8 (20.0%) | 7 (17.5%) |
| Adjuvant radiotherapy | 3 (6.2%) | 2 (5.0%) | 4 (10.0%) |
| Any recurrence | 2 (4.2%) | 15 (37.5%) | 8 (20.0%) |
| Local recurrence | 2 (4.2%) | 13 (32.5%) | 8 (20.0%) |
| Distant recurrence | 0 (0.0%) | 5 (12.5%) | 1 (2.5%) |

**Supplementary Table S2. Recurrence patterns and timing (pT1 only).**

Overall, 25/128 patients (19.5%) developed recurrence. Median time to recurrence was 15 months (IQR 9-22).

| Recurrence pattern | n (%) | Median time to recurrence, months (IQR) |
| --- | --- | --- |
| Local only | 19 (14.8%) | 12 (6-21) |
| Distant only | 2 (1.6%) | 27 (15-15) |
| Local + distant | 4 (3.1%) | 20 (15-21) |

**Supplementary Table S3. Post-TEM management among pT1 patients and associated outcomes.**

| Management | n | Recurrence n (%) | Local recurrence n | Distant recurrence n | Median FU months (IQR) |
| --- | --- | --- | --- | --- | --- |
| No additional treatment | 43 | 6 (14.0%) | 5 | 2 | 24 (24-69) |
| Completion surgery | 17 | 5 (29.4%) | 4 | 2 | 24 (21-63) |
| Recommended completion surgery but not performed | 59 | 11 (18.6%) | 11 | 1 | 28 (24-57) |
| Adjuvant radiotherapy | 9 | 3 (33.3%) | 3 | 1 | 84 (28-118) |

**Supplementary Table S4. Sensitivity analysis by period (pre-2010 vs 2010 onwards) among pT1 patients.**

|  | Pre-2010 | 2010 onwards |
| --- | --- | --- |
| Patients, n | 49 | 79 |
| Recurrences, n (% ) | 7 (14.3%) | 18 (22.8%) |
| Follow-up (months), median | 48 | 24 |

In a multivariable Cox model for DFS adjusting for sm2-3, G3, lymphovascular invasion, completion surgery, and adjuvant radiotherapy, treatment period was not independently associated with DFS: post-2010 vs pre-2010 HR 1.79 (95% CI 0.67-4.77), p=0.242.
